# Cost-Sensitive Decision Support for Industrial Batch Processes

**DOI:** 10.3390/s23239464

**Published:** 2023-11-28

**Authors:** Simon Mählkvist, Jesper Ejenstam, Konstantinos Kyprianidis

**Affiliations:** 1 Kanthal AB, 73427 Hallstahammar, Sweden; 2 Future Energy Center, Mälardalen University, 72123 Västerås, Sweden

**Keywords:** Batch Data Analytics (BDA), feature-oriented, cost-sensitive learning, decision support, machine learning

## Abstract

In this work, cost-sensitive decision support was developed. Using Batch Data Analytics (BDA) methods of the batch data structure and feature accommodation, the batch process property and sensor data can be accommodated. The batch data structure organises the batch processes’ data, and the feature accommodation approach derives statistics from the time series, consequently aligning the time series with the other features. Three machine learning classifiers were implemented for comparison: Logistic Regression (LR), Random Forest Classifier (RFC), and Support Vector Machine (SVM). It is possible to filter out the low-probability predictions by leveraging the classifiers’ probability estimations. Consequently, the decision support has a trade-off between accuracy and coverage. Cost-sensitive learning was used to implement a cost matrix, which further aggregates the accuracy–coverage trade into cost metrics. Also, two scenarios were implemented for accommodating out-of-coverage batches. The batch is discarded in one scenario, and the other is processed. The Random Forest classifier was shown to outperform the other classifiers and, compared to the baseline scenario, had a relative cost of 26%. This synergy of methods provides cost-aware decision support for analysing the intricate workings of a multiprocess batch data system.

## 1. Introduction

The smart manufacturing paradigm is instrumental in fostering a holistic approach to data analytics, wherein the interconnectedness and interdependencies within the manufacturing ecosystem are thoroughly considered. By integrating a plethora of manufacturing agents, it constructs a comprehensive data framework that encapsulates both product specifics and machinery dynamics, thereby enabling analytical interoperability. These integrated data are then processed through a harmonised suite of preprocessing techniques, predictive algorithms, and decision support systems, facilitating the synthesis of this interconnected data into actionable insights.

Furthermore, an interconnected factory facilitates the consistent and systematic analysis of information from individual sections or across the entire plant-wide system. This enables analytics to transcend the cause-and-effect relationships of isolated processes and to encompass plant-wide phenomena. In essence, it allows for an exploration of how product characteristics and sensor data from initial production units impact subsequent processes and final product quality.

Extracting actionable insights from a plant-wide interconnected dataset poses significant challenges. Industrial process data inherently comprise multivariable, non-linear, and frequently, non-Gaussian characteristics, complicating standard analytical approaches [1]. The complexity is further accentuated in batch processing scenarios, which differ markedly from continuous operations. Batch processes are inherently dynamic and non-stationary, introducing variability, which traditional methods are ill-equipped to handle [2]. This complexity underscores the necessity of developing sophisticated decision support systems that are capable of deciphering and acting upon the intricate characteristics present within such datasets.

Additionally, the batch process industries, particularly those engaged in producing specialised products and adapting to mass customisation trends, face exacerbated complexities. These complexities pose significant obstacles in the effective implementation of decision support systems. The crux of the challenge lies in developing a normalised production procedure that is versatile enough to manage a diverse range of products, each with its unique composition, while simultaneously upholding stringent quality tolerances. This task is not only demanding, but also critical in ensuring the adaptability and efficiency of the production process in such dynamic environments.

Furthermore, a legacy industrial batch process often lacks labelled or contextualised data. Data contextualisation in the batch process setting involves connecting a batch to a relevant manufacturing process, that is ensuring the sensor data streams have associated time stamps labelling each batch to its corresponding duration [3]. Correct context is essential. Otherwise, the process parameters are disconnected from the correct batch.

Research in the batch process industry domain frequently utilises Batch Data Analytics (BDA) methodologies. BDA categorises batch process data into three distinct groups: initial properties, variable trajectories, and final properties. The initial and final properties encompass data pertaining to the batch before and after processing, respectively. Meanwhile, data acquired during processing, such as sensor profiles, are classified under variable trajectories.

BDA is adept at handling the unique characteristics of the domain. One such idiosyncrasy is the variability in the time required to produce each batch, leading to differing batch durations. Consequently, the time series data from the batch process inherently produce uneven datasets. Therefore, special attention is necessary to effectively manage these variable trajectories in conventional analytics.

To that end, BDA presents feature generation to align the variable trajectories. Feature generation creates statistics from time series data, resulting in an aligned dataset. A variable trajectory transformed using feature generation is called a variable feature. The feature landmark approach, introduced by Wold et al. [4], is an example of feature generation. A derivation of the feature landmark approach, called Statistical Pattern Analysis (SPA) feature generation, was used and elaborated by Wang and He [1], He and Wang [5], He et al. [6]. Two other feature-generation approaches may also be observed: profile-driven features Rendall et al. [7] and translation-invariant multiscale energy-based features Rato and Reis [8].

Another method of BDA feature accommodation approaches the problem by aligning the batch data and is called called trajectory alignment. As the name implies, trajectory alignment warps the trajectory of the batches to a standard duration and, depending on the complexity of the method, synchronises distinct characteristics such as maxima and minima. Notable works on batch data transformation using trajectory alignments are Nomikos and MacGregor [9,10,11].

A machine learning predictive model will serve as the cornerstone of the decision support system, offering probabilistic insights. Utilising supervised machine learning, it is possible to forecast the final properties of a batch process dataset, drawing on initial features and/or variable features as inputs. Supervised learning tasks can manifest as either regression or classification challenges. The key distinction lies in regression predicting quantitative outcomes, while classification focuses on qualitative ones. Additionally, there exists a specific scenario in classification with only two possible outcomes, such as ‘true’ or ‘false’, known as binary classification [12].

Implementing and comparing various machine learning methods is prudent since no method is inherently superior to another on an unseen dataset [12]. Additionally, implementing models in increasing order of complexity lends to a parsimonious approach. Striving towards suitable modelling and implementation complexity is beneficial. It reduces the time it takes to analyse the problem and makes it perceptible [2]. Various models can capture different aspects and characteristics of the process.

In this work, machine learning will be used in the context of batch data to determine if the batch will comply with final property tolerances. The problem was designed as a binary classification problem, determining if the batch is predicted to be compliant or non-compliant. An assortment of classification models will be used to achieve the best-possible accuracy in this scope.

A decision support system was constructed using the classifier. The accuracy metric was used for the model’s confidence and was derived from the confusion matrix. The confidence of the classifiers was used as an intervention threshold for when to utilise the model’s recommendation for decision support. The threshold effectively acts as a filter, discarding samples whose confidence metric is below the threshold. As a result, increasing the lowest allowed confidence reduces the coverage of the model, i.e., the out-of-coverage samples do not receive a recommendation from the decision support system.

For the cost sensitivity analysis to be pertinent, it is imperative to make accommodations for out-of-coverage individuals (called transgressors). Otherwise, the analytics loses relevance with reality. An adjustable probability threshold gives control over the decision support’s accuracy. This control is appealing because it can be configured to conform to the level of uncertainty acceptable by the business. Hence, there is a need to consider the transgressors. Two transgressor strategies’ are presented and compared to discover the most cost-efficient approach. One strategy accepts the transgressors and sends them for further processing; the other strategy rejects them and sends them to be scrapped.

Cost-sensitive learning is incorporated into the decision processes to optimise the net savings of the decision support system. Incorporating the associated mislabelling costs into the modelling and decision support is called cost-sensitive learning, and it is implemented by developing a cost matrix. The cost matrix complements the confusion matrix, but instead of illustrating the false-positive frequency, it depicts the false-positive costs. In other words, the cost matrix displays the corresponding cost of erroneously labelling individuals. The concept of cost-sensitive learning and the cost matrix was introduced by Elkan [13]. Other notable works on cost-sensitive learning can be found in Gan et al. [14] and Verbeke et al. [15].

Cost-sensitive learning stands out as an essential technique in machine learning, especially for addressing the complexities associated with imbalanced datasets and varying misclassification costs. As detailed in the works of Ghatasheh et al. [16], Lobo et al. [17], cost-sensitive learning adeptly incorporates cost considerations into the training phase of machine learning models. This is typically achieved through internal re-weighting of dataset instances or by adjusting misclassification errors, which allows for the construction of a prediction model that prioritises lower-cost outcomes over higher probability ones.

This approach is particularly beneficial in scenarios with class imbalance and significant misclassification costs, as emphasised by Zhang et al. [18] in the context of manufacturing industries. Here, cost-sensitive learning is instrumental in tailoring decision support systems to focus not just on accuracy, but also on cost effectiveness, demonstrating its versatility and efficacy in diverse applications.

The authors found tangential research in several domains. Alves et al. [19] studied stabilising production planning. However, instead of focusing on the product’s compliance and the associated classification cost, they focused on failure prediction in industrial maintenance in the context of tardiness. Additionally, the general conclusions were that the managerial role is shifting from problem-specific to being able to develop different strategies for various scenarios, such as the solution presented in this work.

Further, Frumosu et al. [20] worked on predictive maintenance with an imbalanced classification dataset and incorporating cost sensitivity. In the context of the multi-state production of frequency drivers, given historical quality data, they predicted the final product quality before shipping to the client.

Next, the work of Verbeke et al. [21] analysed the impact of following prognostic recommendations. They designed a decision support model. The concept of an individual treatment effect is similar to the cost-sensitive approach in this work, where the individual treatment effect looks at the difference between the outcome with and without applying the effect.

Additionally, Mählkvist et al. [22] considered using industrial batch data analysis with machine learning. Here, the industrial batch data were first consolidated using BDA methods to be able to analyse the data with machine learning. Then, the data were explored using the machine learning method kernel-principal component analysis. The results indicated that it is feasible to derive patterns from the batch data trajectories using a combination of SPA (for feature generation) and kernel-principal component analysis.

For a proof of concept, the thermocouple manufacturing process at Kanthal was examined. In this production, thermocouples are created in the form of wire rod coils. Two key production units, the smelting furnace and the wire rod rolling mill, provide input for the decision support system. The wire rod rolling mill, with its complex and unaligned time series data, offers a valuable case for enhancing the decision support system through feature generation. Specifically, this involves integrating and contrasting models that utilise features derived from time series sensor data against those relying solely on product property data. See Section 2.1 for more details.

The thermocouples’ production provides the data for the model. After the smelting furnace, the initial properties data are sampled from the batch’s properties. Next, the variable trajectory data are acquired from the wire rod rolling process. After the wire rod rolling mill, the final properties’ data are sampled from the batch’s properties. A binary classifier was designed to predict if a batch will comply with the property tolerances using properties from earlier in the operations. Three classification methods were implemented and reviewed with respect to the model accuracy. The classification models were Logistic Regression (LR), Random Forest Classification (RFC), and Support Vector Machine (SVM).

This paper integrated a constellation of methods and approaches from the different fields of BDA, cost-sensitive learning, and machine learning to optimise operational decision-making for an industrial batch process from a cost-aware perspective. The model acts as a decision support system to prevent redundant processing by salvaging the batch before additional processing, that is preventing superfluous investment. Cost-sensitive learning, machine learning methods, and BDA were combined to create a cost-sensitive decision support system. The model accuracy and the relative cost were compared by subjecting the case study to different scenarios, that is, modelling with and without the trajectory features and varying the two transgressor strategies, accepting or rejecting out-of-coverage batches.

The paper is structured as follows: In Section 2, the methodology is described. In Section 3, the results are presented and subsequently discussed. Next, in Section 4, the details of the paper are synthesised into conclusions.

## 2. Methodology

### 2.1. Case Study: Thermocouple Alloy Manufacturing

**Figure 1:**
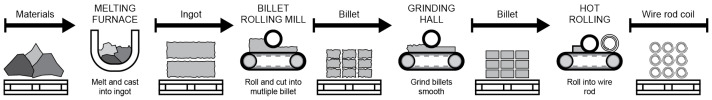
Illustration of the manufacturing process.

The system under observation manufactures thermocouple alloys in the format of wire rods. The system flowchart is illustrated in Figure 1, and a manufacturing procedure composed of multiple processes is explained in this figure. The batch originates from the melt shop, where different raw materials are melted, processed, and finally, cast to produce one or more ingots. The batch (now separated into multiple sub-batches) is then compressed by rollers into a smaller square cross-section and cut into billets of adequate lengths in the billet rolling mill. Subsequently, the billets are ground smooth in the grinding hall and rolled into wire rod coils in the hot rolling mill.

#### Properties and Sensors

The evaluation of a thermocouple primarily relies on the Electromotive Force (EMF) it generates. In this study, EMF measurements were taken for both the initial ingot and the subsequent wire rod batches. These measurements were conducted across a range of temperatures, from which two specific temperatures were identified as crucial. These temperatures were selected based on the observation that deviations at these points typically indicate subsequent tolerance breaches in the entire process. The EMF measurements at these critical temperatures are designated as $E_{1}$ and $E_{2}$. Additionally, the chemical composition of each ingot batch was meticulously analysed, focusing on nine specific elements, labelled as $C_{1}$ to $C_{9}$.

The process begins with the ingot produced from the melting furnace, where its EMF values and chemical composition are recorded. Subsequently, EMF measurements are also performed on the wire rod coils. During the wire rod rolling phase, sensor data capturing the temperature, speed, and power are collected and analysed. This study compiled and examined data from 119 melt batches, which encompassed a total of 1049 wire rod sub-batches, to derive insights and correlations.

### 2.2. Consolidating Batch Data

**Figure 2:**
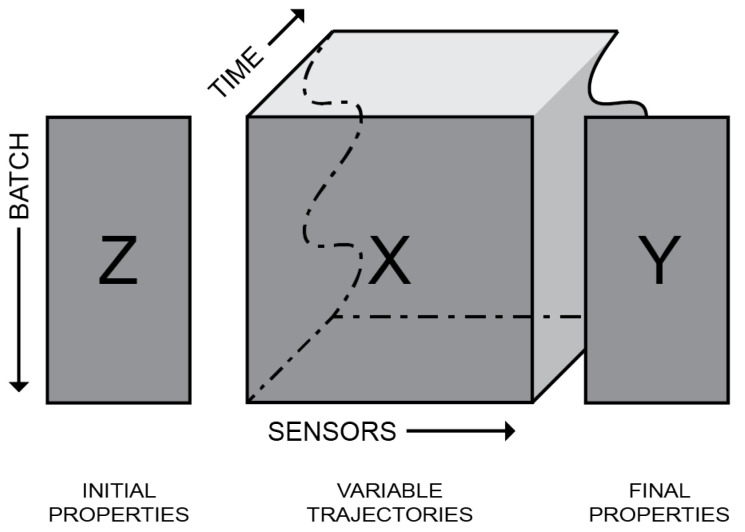
Batch data structure.

BDA provides a systematic approach to analyse batch process data. When implementing the taxonomy introduced by the work of Wold et al. [4] on batch data analysis, a batch process dataset can be categorised into three parts: initial properties, variable trajectories, and final properties, for which the following symbols are used, respectively, Z, X, and Y. Figure 2 illustrates the taxonomy and data structure relevant to interpret the batch data concept. Hence, the three different segments of the batch processes datasets are illustrated. In this illustration, the height represents the number of batches, where each row is a specific individual. Further, width signifies the number of features, where each column is a specific property or variable. The initial properties and the final properties are two-dimensional datasets, where the variable trajectory consists of three dimensions. The variable trajectories are time series sensor measurements whose duration is depicted by adding depth. Further, due to the batch duration’s inherent variation, an undulating line is used to depict the inconsistency.

Each batch is split into multiple wire rod sub-batches at the billet rolling mill. Therefore, there is a one-to-many relation between the ingot batch and the wire rod sub-batch. In practice, this means that the ingot data are duplicated over the corresponding wire rods’ data.

#### 2.2.1. Feature Generation

The variable trajectories require deliberate consideration to align with the initial and final properties. i.e., converting the three-dimensional variable trajectories into two-dimensional variable features. To this end, Statistical Pattern Analysis (SPA) was used. SPA is a feature-generation approach that isolates individual time series and extracts statistics. The derived statistics, called variable features and denoted **X_f_**, are derived from the variable trajectories. The statistics used were:

**Mean:** this is the value of the centre of the distribution.
**Skewness:** this signifies the asymmetry of the distribution with respect to its mean.
**Kurtosis:** a measure that tells about the spread of the data and how spread the distribution is from the mean.

Rarely does an industrial batch process conform to a Gaussian distribution, and to that end, skewness and kurtosis were used by [5].

**Figure 3:**
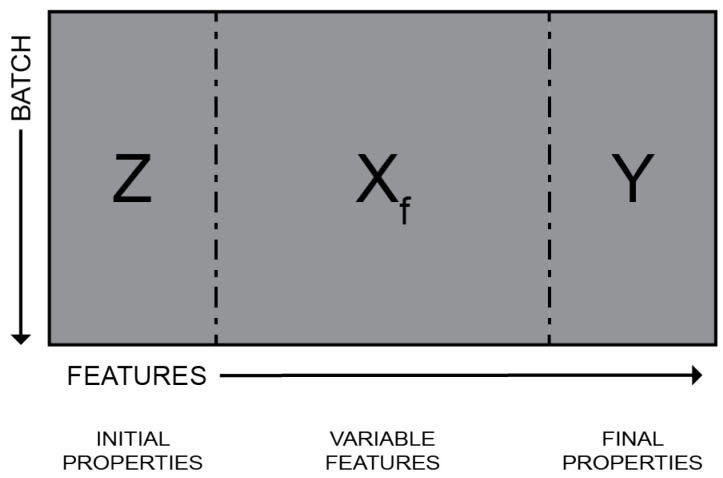
Unfolded batch data.

A feature-oriented approach allows the data segments to be concatenated batchwise. The variable trajectories are flattened and aligned by converting variable trajectories (time series) into variable features (statistics), as illustrated in Figure 3. Further, this illustration depicts the unfolded transformation of the batch data structure. The variable trajectories were converted to variable features and aligned batchwise with the initial and final properties.

#### 2.2.2. Preprocessing

In the preprocessing phase, the features of the unfolded batch data were initially filtered using a variance filter. Subsequently, the data were standardised to attain a variance of 1 and a mean of 0. Two distinct datasets were then prepared: one incorporating the variable features and another excluding them. The purpose of this comparative analysis was to determine whether the variable trajectories, derived from the hot rolling processes, influenced the final properties of the datasets.

### 2.3. Classification Models

Classification is one of two supervised learning approaches; the other is regression. Classification operates in a qualitative setting, whereas regression does so quantitatively. The accuracy metric defines the goodness of fit for classification models. The accuracy metric informs about the ability of the model to accurately predict the true positives and true negatives out of the population. When the response of a qualitative dataset has two classes, it is called binary classification.

The reason for using logistic regression, SVM, and RFC in parallel is that they are of different levels of complexity and are members of two different types of methods. Namely, LR and SVM are parametric methods, whereas RFC is non-parametric. The parametric approach assumes that a set of parameters can interpret any perceived patterns of the features against the response. Contrarily, the non-parametric approach does not assume a structure and tries to fit the features against the response whilst satisfying some goodness of fit criteria.

Implementing models of incremental complexity is beneficial because of the relative ease of modelling and implementation compared to models of higher complexity. The models’ can interpret the relationship between the features and response with varying levels of flexibility, but increased flexibility results in greater complexity. In this study, LR is an example of a model with low flexibility and complexity, whereas SVM and RFC have comparatively high flexibility and complexity [12]. Adhering to the principle of parsimony, there is no justification for employing a high-complexity model when a low-complexity model suffices for the intended purpose, as complexity alone does not constitute a sufficient rationale.

In this study, the random search method was employed for hyperparameter estimation. This technique involves the random selection of hyperparameters from a predefined range of possible values. One of the key advantages of random search is its reduced computational demand, especially in comparison to more-traditional, exhaustive search methods. Despite its lower resource requirements, random search often yields performance that is on par with, or even superior to, these exhaustive approaches. This efficacy was highlighted in the work of Bergstra and Bengio [23], who demonstrated the efficiency and effectiveness of random search in hyperparameter optimisation, particularly in scenarios where the search space is large or the relationship between hyperparameters and model performance is not straightforward. This makes random search an appealing choice for efficiently navigating complex hyperparameter landscapes.

Each model possesses distinct hyperparameters, which are detailed in their respective sections. Moreover, a comprehensive list of all classifier and dataset combinations is provided in the accompanying Table 1. Two models were created per classifier, one for each dataset, making a total of six models. The two datasets were the initial properties (**Z**) and the initial properties with variable features (**X_f_**). To make sense of the model labelling convention, the classifier abbreviations denote the deferred model, and the dataset abbreviation denotes the deferred dataset in the subscript. When the initial properties and variable features are combined, **X** is used. The classification models in this study were developed using the scikit-learn Python package (version 1.3.0)) [24]. The following sub-section will detail the estimation of the classification models.

#### 2.3.1. Logistic Regression

Logistic regression is a classification method known by other names: logit regression, log-linear classifier, and maximum entropy classification. Logistic regression is used as a baseline approach (from a modelling complexity perspective) in this binary classification problem due to its fundamental nature.

In binary classification, out of two possible classes, logistic regression models the probability that a sample is a member of one class.

Logistic regression works by estimating the logistic model parameters, and the maximum likelihood method is used to estimate the hyperparameters of the logistic model [12].

The logistic regression parameter is the inverse strength of regularisation (denoted as C). For regularisation, the L2-norm is applied.

#### 2.3.2. Random Forest

The Random Forest algorithm determines its predictions for classification tasks based on the majority vote from its ensemble of decision trees. An array of decision trees is trained, and then, they place their vote; as a result, the most-popular class is the resulting prediction. The trees are all derived from the same distribution. Random Forest is insensitive to over-fitting. Random Forest is an ensemble of tree predictors, and in the case of the Random Forest classifier, the trees are classification decision trees. The trees are grown using recursive binary splitting. In the case of classification, each observation is assigned to the most-prevalent class of the training region [25]. The Gini index is used to compute the impurity of the region and measures the total variance for the classes [12].

The modelling parameters are the number of trees to use in the forest, the maximum allowed tree depth, applying bootstrapping, the minimum sample for a leaf, the minimum samples for a split, and the number of features to carry over.

#### 2.3.3. Support Vector Machine

SVM builds on its precursor, the support vector machine classifier, by using kernels to expand the feature space, which accommodates non-linear characteristics [12].

Three different kernels are used: linear, polynomial, and Radial Basis Function (RBF). The modelling complexity increases, respectively. The most-accurate hyperparameter setup will be presented. Multiple modelling hyperparameters can be adjusted for greater accuracy. All kernels adjust the inverse strength of regularisation, denoted as C, which is the linear kernel’s sole parameter. The kernel coefficient γ is used by both the polynomial and RBF kernel. The polynomial kernel has a degree parameter to determine which degree of polynomial to use.

### 2.4. Cost-Sensitive Decision Support

This section outlines a methodology for incorporating cost considerations into the predictive model. It also evaluates the costs associated with employing these models in a decision support system compared to a baseline scenario. In this baseline scenario, all batches are presumed to be compliant, representing a default cost strategy. However, since the actual cost data are proprietary to Kanthal, we used relative cost values for the analysis. To demonstrate the effectiveness of the decision support system, it must offer a cost advantage over the baseline. That is, the costs incurred under the baseline scenario should not exceed those when adhering to the system’s recommendations.

The confusion and cost matrices illustrate the accuracy and cost associated with using the model for decision support. The matrices reveal metrics for the classification, and where the confusion matrix shows the population count, the cost matrix contains the misclassification cost [13].

Let N and C be the confusion and cost matrix, respectively. N is shown in Table 2. C is illustrated using the structure seen in Table 3. Both the confusion matrix and the cost matrix adhere to the convention of utilising rows to represent the predicted class and columns to indicate the actual class. To make the terminology more intuitive and aligned towards the case study, the predicted true and false naming convention is called accept and reject, as the recommendation is to accept or reject the batch. For the resulting class, the true and false outcomes are termed ‘compliant’ and ‘non-compliant’, respectively. This categorisation reflects whether a batch falls within or outside the specified tolerance.

Further, the cost matrix focuses on the expense of misclassification. Therefore, the cost matrix has no associated cost for correct classifications. Also, the cost of mislabelling a batch should always be higher than labelling it correctly. Additionally, the cost matrix can be transformed by adding or multiplying the matrix with positive constants [13]. Hence, the actual cost can be anonymous and relevant using the relative cost. The relative cost is derived by dividing the cost matrix by the highest cost of the cost matrix, i.e., the cost of rejecting a batch that would have been compliant.

(1) $$\begin{array}{c} S\left(i,j\right)=\sum_{j}^{2}\sum_{i}^{2}C(i,j)N(i,j)\\ =c_{\left(i=0,j=0\right)}n_{\left(i=0,j=0\right)}+c_{\left(i=0,j=1\right)}n_{\left(i=0,j=1\right)}\\ +c_{\left(i=1,j=0\right)}n_{\left(i=1,j=0\right)}+c_{\left(i=1,j=1\right)}n_{\left(i=1,j=1\right)} \end{array}$$

Equation (1) expresses how to calculate the total cost using a confusion matrix and a cost matrix, where *S* is the total cost. The predicted and actual classes are *i* and *j*, respectively.

#### 2.4.1. Accuracy–Coverage Trade-Off

The classifier labels batches as either accept or reject. Each sample has a corresponding accuracy metric and probability estimation, showing the discriminatory power of the classifier for that batch. The probability estimation is utilised to filter weak predictions; a probability threshold is determined, and if the batch falls under the threshold, it is labelled a transgressor. Increasing the probability threshold results in a lower proportion of individuals being subjected to decision support, i.e., the number of samples in which the model can provide support is reduced. This is termed as coverage. As a result, the coverage of the model decrease. But, since the minimum accuracy is increasing, it results in overall greater accuracy.

#### 2.4.2. Transgressor Accommodation

Transgressors, identified as out-of-coverage samples that do not meet the set probability threshold, still bear associated costs. It is recommended that the decision support system utilises this probability threshold as a guide to selectively consider the model’s predictions. For effectively managing the material and processing costs related to these transgressors, two specific strategies should be implemented. The first, an open approach, involves continuing the processing of transgressors. The second, a closed approach, recommends the rejection of transgressors. These strategies are intended to optimise resource allocation and minimise unnecessary expenditures.

## 3. Result and Discussion

### 3.1. Preprocessing and Class Balance

**Figure 4:**
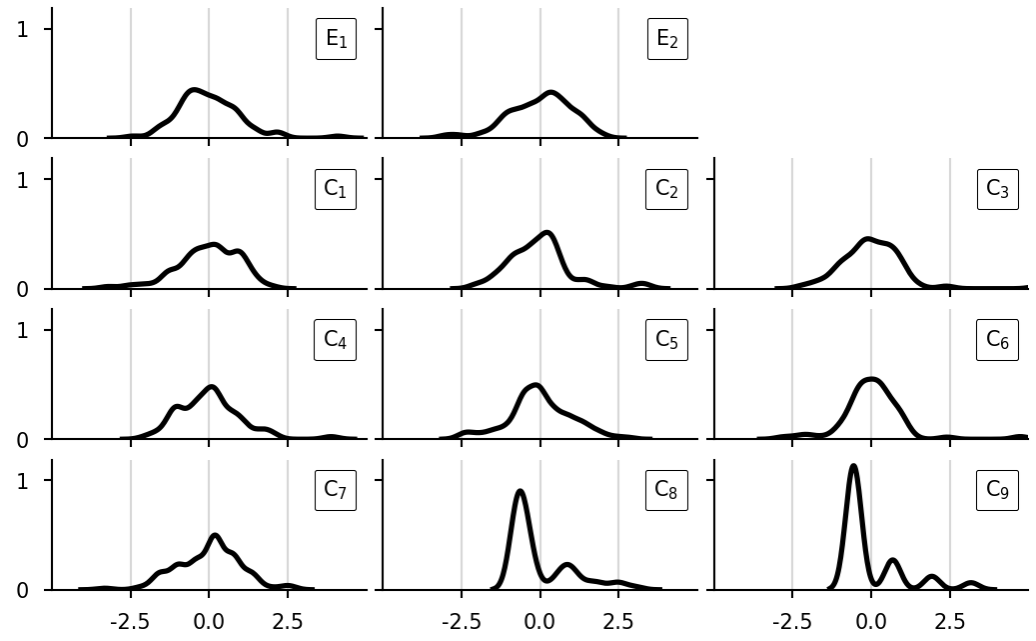
Initial features.

The normalised distribution of the initial properties (containing the EMF-values and chemical composition) is plotted in Figure 4. The figure’s subfigures share the x- and y-axis limits and resolution. The two subfigures in the top row display the two EMF values ($E_{1}$ and $E_{2}$). The remaining subfigures in the second to the last row show the content of nine chemical elements ($C_{1}$ to $C_{9}$).

The last two chemical properties $C_{8}$ and $C_{9}$ in Figure 4 are noteworthy. Compared to the other properties, they diverged from the normal distribution. Further, it is discernible that they were sampled at a coarser resolution. Also, both skew to the left. Other than this, the initial properties are observably normally distributed.

**Figure 5:**
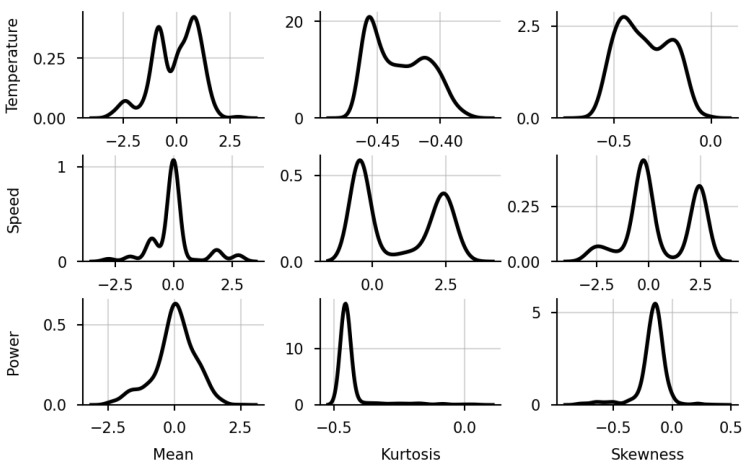
Variable features.

The variable features (transformed from variable trajectories) are seen in Figure 5. Using SPA, these features were generated from normalised sensors’ time series data for each wire rod sub-batch. The three rows of the figure correspond to sensors registering the following: temperature, speed, and power. The three columns of the figure represent the three statistics: mean, kurtosis, and skewness.

The skewness and kurtosis of the speed feature show two different clusters, which are not discernible over the speed mean—indicating that the process operates at two separate distributions. These clusters are also identifiable in the temperature’s kurtosis and skewness, and their two peaks are also seen in the mean.

**Figure 6:**
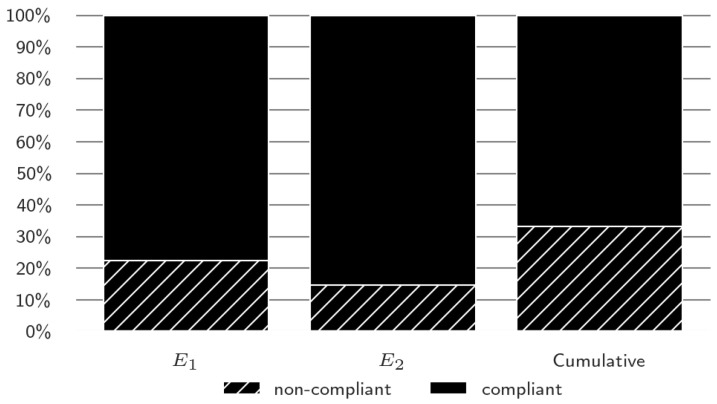
Final properties’ class distribution: individual and cumulative.

The class distribution is depicted in Figure 6. It shows a union of non-compliant batches over the final properties ($E_{1}$ and $E_{2}$); this can be observed in the figure’s left and centre columns. Multiple batches are non-compliant only in the individual properties. The total of non-compliant individuals and the overlap between the properties are seen in the right bar of Figure 6 labelled cumulative.

The ratio of non-compliant batches for $E_{1}$ and $E_{2}$ was 22.4% and 14.7%, respectively. Further, the ratio of the non-compliant batches for the cumulative was 33.2%. As stated, the cumulative is not the sum of $E_{1}$ and $E_{2}$ since there is an overlap. The ratio of binary classes for the cumulative class was sufficiently high not to have to implement methods for imbalanced classification.

### 3.2. Cost and Confusion Matrix

The cost matrix (seen in Table 4) was implemented for all models under observation (see Table 1). There is no cost associated with rejecting a non-compliant batch. Likewise, there is no cost to accepting a compliant batch. Hence, $c_{00}=c_{11}=0$. The cost of rejecting a compliant batch corresponds to the material and processing expenses of producing an ingot. The cost was estimated to 75% of the total cost, i.e., $c_{01}=75$. Further, the cost associated with accepting a non-compliant batch accumulates the material and processing costs of the entire scope, from the melting furnace to the hot rolling mill. Hence, 100% of the cost and $c_{10}=100$.

The confusion matrix for the baseline scenario is shown in Table 5. In this scenario, the individuals were distributed between accepting compliant and accepting non-compliant. The batches used were all deemed acceptable within the scope of the operation; hence, there were no rejected batches in the baseline case. The proportion of accepted compliant and non-compliant individuals was 60 and 40%, respectively.

### 3.3. Hyperparameter Estimation

The scope of the hyperparameter search is shown for LR, RFC, and SVM in Table 6, Table 7, and Table 8, respectively. Also, these tables show the parameters for the best cross-validated models with and without the variable features.

LR’s sole parameter, C, was 10 and 0.1 for LR_X_ and LR_Z_, respectively. The two SVM models showed a slight disparity. SVM_X_ had a lower C, but a higher degree parameter compared to SVM_Z_. It can be observed that SVM_X_ had a lower C than SVM_Z_, which is the opposite relationship, considering the datasets shown for LR.

For the RFC models, there was a clear difference between RFC_X_ and RFC_Z_, considering the complexity of the parameters. RFC_X_ had higher values in the number of trees and the maximum tree depth. This difference implies that RFC_X_ works towards consolidating the complexity added by the variable features since both the number of trees and the maximum tree depth increase flexibility. The rest of the features were the same for both models.

### 3.4. Classification Accuracy–Coverage Trade-Off

**Figure 7:**
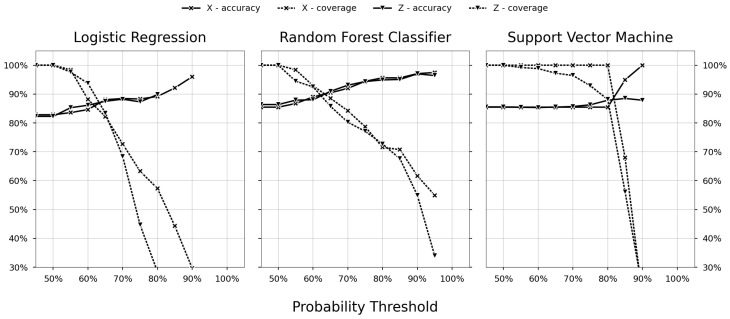
Accuracy versus coverage trade-off.

The hyperparameters were used to train a classifier and subsequently implemented as a foundation for the decision support model. The accuracy and coverage of the model were calculated over an incrementally increasing probability threshold from 50–100%. In Figure 7, the trade-off between the accuracy and coverage is shown, that is the impact of increasing the probability threshold as the coverage decreases, while the accuracy increases for the three different classifiers: LR (left figure), RFC (centre figure), and SVM (right figure). The limits of the x- and y-axis are shared between the sub-figures. The y-axis shows the value of any of the metrics as a percentage, and the x-axis is the probability threshold as a percentage. Each figure contains the accuracy and coverage for the two datasets, with and without variable features, separated by different markers. For the dataset using feature variables, an x-shaped marker is used. For datasets without feature variables, a down-pointing triangle is used. Further, the accuracy and coverage are separated by line style, where the former is solid and the latter is dashed.

LR insinuates the least-profitable relationship between coverage and accuracy, indicating that the underlying behaviour cannot be adequately interpreted with that degree of complexity. The accuracy and coverage of RFC and SVM seem comparable, and both outperformed LR. There was a conspicuous behaviour in SVM_X_. The accuracy and coverage trade-off of SVM_X_ were inconsistent with the other models, showing a sharp decrease in accuracy. The reason may be that the classifier performs an excellent job in separating the clusters. That is, samples in closer proximity to the separating hyperplane act as support vectors or are within the decision boundary. Further, seeing as the regularisation parameter is quite strict (C=0.01), it makes sense to see a sharp cut-off.

RFC had the most-reliable trade-off between accuracy and coverage. While RFC_X_ had slightly better accuracy compared to RFC_Z_, they performed comparatively equally. There was no strong indication that either RFC model would outperform the other, i.e., the variable features provided no apparent benefit over just using the initial features.

### 3.5. Relative Cost and Transgressor Accommodation

**Figure 8:**
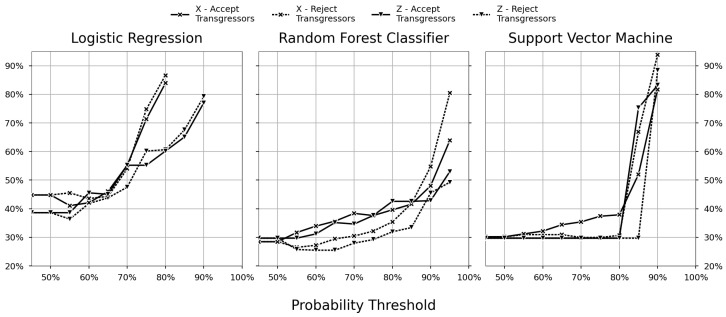
Relative cost.

The impact of the accuracy–coverage trade-off on the cost is elaborated in Figure 8, which also compares the two cost scenarios for dealing with transgressor batches. The figure shows the relative cost against baseline operations for three different classifiers: LR (left figure), RFC (centre figure), and SVM (right figure). Each figure shows four cases comparing the two datasets, with and without sensors data, and the two transgressor scenarios, accepting or rejecting the batch. The relative cost was calculated using Equation (1) for each step in the probability threshold and dividing it using the same equation, but for the baseline scenario.

For the dataset using feature variables, the x-shaped marker is used. For datasets without feature variables, a down-pointing triangle is used. Further, transgressor scenarios are separated by line style, where accepting and rejecting the transgressor has solid and dashed line styles, respectively. Additionally, each sub-figure shares the limits and resolution of the x- and y-axis. The y-axis shows the relative cost, as a percentage, of the different cases, and the x-axis is the probability threshold as a percentage.

The weak accuracy shown for LR in Figure 7 was carried over to the relative cost and results in the poor performance in both transgressor scenarios. In Figure 8, it can be observed that LR achieved a minimum cost by increasing the probability threshold to 55%. The model that achieved this was LR_Z_ with the rejection scenario, which did not contain the variable features, i.e., the least-complex dataset. Beyond the 55% probability threshold, the relative cost for LR_Z_ with the rejection scenario suffered from diminishing returns.

The peculiar behaviour of the SVM models previously observed in the accuracy coverage trade-off in Figure 7 is visible for the relative costs in Figure 8. Further, only the SVM_X_ model with the reject transgressor scenario improved with an increased probability threshold.

While RFC and SVM seemed equivalent regarding accuracy and coverage, it is discernible in Figure 8 that RFC performed best. RFC achieved optimal cost savings relative to the base case when it was at 65%. At this probability threshold, the RFC_Z_ model with a reject transgressor cost scenario achieved a relative cost of 26% (compared to the baseline). After this point in the probability threshold, the model suffered from diminishing returns on the relative costs.

## 4. Conclusions

The current study successfully showed that an integrated approach, combining BDA, machine learning, and cost-sensitive learning, plays a pivotal role in reducing overall costs. This synergy effectively aids in determining the most-advantageous discrimination settings, optimising both accuracy and cost efficiency. By leveraging the strengths of each component, this combined methodology presents a comprehensive solution for efficient decision-making in complex scenarios.

Two methods from BDA were implemented, the batch process data structure, which organises the data into initial properties, variable trajectories, and final properties, and the feature accommodation approach SPA, which transforms variable trajectories into variable features. These two methods resulted in an aligned two-dimensional dataset primed for use with machine learning. To determine if the variable features improved the prediction accuracy, the machine learning models were trained both with and without these, and the result was reviewed.

Three machine learning classification methods were implemented and reviewed: logistic regression, Random Forest classification, and support vector machine. These cover an increasing level of complexity where logistic regression is the least complex and Random Forest and support vector machines are about equal. Random Forests and support vector machines differ in that the former are non-parametric and the latter parametric, allowing them to interpret different characteristics.

After training the models to achieve optimal accuracy, a probability threshold was introduced. This threshold increased from 50 to 100%, and the filtered out batches met the criteria of the probability limit. Consequently, this evolved into an accuracy–coverage trade-off in which a stringent probability threshold increased the accuracy and reduces coverage. The accuracy–coverage trade-off did not provide an optimal solution, and to this end, the cost matrix can be leveraged.

Cost-sensitive learning introduces the cost matrix. The cost matrix determines which probability threshold provides the optimal relative cost. The accuracy–coverage trade-off can be interconnected with the associated relative costs. Further, the flexibility to configure the probability to suit a risk is deemed acceptable by the industry. This work also improved the relative cost of a case study using the aforementioned decision support system.

The optimal cost-efficient solution was RFC_Z_ in the reject transgressor scenario. The Random Forest classifiers only use the initial features as the input data. The best probability threshold was at 65% and resulted in a relative cost of 26%.

When comparing the models using a dataset with or without variable features (aggregated sensor data), the impact that the variable features had on the accuracy was negligible or even detrimental (as one can observe in the accuracy–coverage trade-off in Figure 7). In particular, models trained with variable features performed inferior to those without. The possible causes can be as follows:

- Despite the variance in the variable features (seen in Figure 5), the models discerned no correlation between the process and the response;
- The statistics used (mean, skewness, and kurtosis) were not sufficient to interpret the features’ intrinsic behaviour;
- The classifiers were not flexible enough to interpret the relationship between the features and response.

There are other metrics to consider besides the relative cost. It may be that the operation needs to optimise against quantity rather than quality. Hence, depending on the need of the system, it may be beneficial to tighten or lessen the probability constraint. But, having a sparse strategy provides other benefits, such as: lowering the strain on down-stream production, reducing the time for quality products to be produced by cutting the process short for sub-par products, and higher quality stock.

Another aspect that could improve the decision support system is to expand the definition of non-compliant batches. A non-compliant individual may match another non-compliant individual that balances out the out-of-bounds EMF; hence, a supplemental model that determines the likelihood of matching other sub-par individuals can be used to capture this. Also, it may be possible to redeem some of the cost for the metallurgical process by re-melting the batch since a scrap value is associated with the batch. Hence, leaving the rejected non-compliant batch cost at zero may not represent the reality for the cost matrix.

In conclusion, this approach provides a cost-sensitive decision support system with accommodation scenarios for out-of-coverage batches. It allows for configurable sensitivity constraints, either based on the needs of the industry or by optimal cost. The accuracy–coverage trade-off illustrates clearly how a stricter threshold results in reduced decision support coverage. While it is infeasible to analyse the optimal cut-off point from the accuracy–coverage trade-off, the relative cost plot is discernible. Thus, the cost-sensitive approach allows the accuracy coverage to be extended to the relative cost in which an optimal value can be identified. This allows for a clear decision about the cut-off point, converting the abstract problem into actionable insights, consequently, creating value from the data.

## Figures and Tables

**Table 1:** Classifiers and dataset abbreviation.

Name	Classifier	Dataset
LR_Z_	LR	**Z**
LR_X_	LR	**Z** & **X_f_**
RFC_Z_	RFC	**Z**
RFC_X_	RFC	**Z** & **X_f_**
SVM_Z_	SVM	**Z**
SVM_X_	SVM	**Z** & **X_f_**

**Table 2:** Confusion matrix (*N*).

	Non-Compliant	Compliant
Reject	$N\left(0,0\right)=n_{00}$	$N\left(0,1\right)=n_{01}$
Accept	$N\left(1,0\right)=n_{10}$	$N\left(1,1\right)=n_{11}$

**Table 3:** Cost matrix (*C*).

	Non-Compliant	Compliant
Reject	$C\left(0,0\right)=c_{00}$	$C\left(0,1\right)=c_{01}$
Accept	$C\left(1,0\right)=c_{10}$	$C\left(1,1\right)=c_{11}$

**Table 4:** Relative cost matrix.

	Non-Compliant	Compliant
Reject	0	75
Accept	100	0

**Table 5:** Baseline confusion matrix.

	Non-Compliant	Compliant
Reject	0	0
Accept	40	60

**Table 6:** Logistic regression hyperparameter estimation: scope and best model settings.

Parameter	Search Scope	Best Model LR_X_	Best Model LR_Z_
C	$10^{n},n=\left[-2,-1,\ldots ,2\right]$	10	0.1

**Table 7:** Random forest classifier hyperparameter estimation: scope and best model settings.

Parameter	Search Scope	Best Model RFC_X_	Best Model RFC_Z_
Number of Trees	200–2000	2000	200
Max Tree Depth	10–100	90	30
Bootstrap	On/Off	On	On
Min Sample Leaf	1, 2, 4	1	1
Min Sample Split	2, 5, 10	2	2

**Table 8:** Support vector machine hyperparameter estimation: scope and best model parameters.

Parameter	Search Scope	Best Model SVM_X_	Best Model SVM_Z_
Kernel	Linear, Polynomial, RBF	Polynomial	Polynomial
Gamma	$10^{n},n=\left[-2,1,\ldots ,1\right]$	1	1
C	$10^{n},n=\left[-2,1,\ldots ,2\right]$	0.01	0.1
Degree	2,3,4	2	3

## Data Availability

The data underpinning this study are confidential and proprietary, exclusive to Kanthal, and therefore cannot be shared publicly. Additionally, this study did not involve the creation or analysis of new data that could be made available for sharing.

## Abbreviations

The following abbreviations are used in this manuscript:

BDA	Batch Data Analytics
C	Cost matrix
EMF	Electromotor Force
LR	Logistic Regression
N	Confusion Matrix
RBF	Radial Basis Function
RFC	Random Forest Classifier
SPA	Statistical Pattern Analysis
SVM	Support Vector Machine

## References

[1] Wang J., He Q.P. (2010). Multivariate Statistical Process Monitoring Based on Statistics Pattern Analysis. Ind. Eng. Chem. Res..

[2] Rendall R., Chiang L.H., Reis M.S. (2019). Data-Driven Methods for Batch Data Analysis—A Critical Overview and Mapping on the Complexity Scale. Comput. Chem. Eng..

[3] Cerquitelli T., Ventura F., Apiletti D., Baralis E., Macii E., Poncino M. (2021). Enhancing Manufacturing Intelligence through an Unsupervised Data-Driven Methodology for Cyclic Industrial Processes. Expert Syst. Appl..

[4] Wold S., Kettaneh-Wold N., MacGregor J., Dunn K. (2009). Batch Process Modeling and MSPC. Comprehensive Chemometrics.

[5] He Q.P., Wang J. (2011). Statistics Pattern Analysis: A New Process Monitoring Framework and Its Application to Semiconductor Batch Processes. AIChE J..

[6] He Q.P., Wang J., Shah D. (2019). Feature Space Monitoring for Smart Manufacturing via Statistics Pattern Analysis. Comput. Chem. Eng..

[7] Rendall R., Lu B., Castillo I., Chin S.T., Chiang L.H., Reis M.S. (2017). A Unifying and Integrated Framework for Feature Oriented Analysis of Batch Processes. Ind. Eng. Chem. Res..

[8] Rato T.J., Reis M.S. (2017). Multiresolution Soft Sensors: A New Class of Model Structures for Handling Multiresolution Data. Ind. Eng. Chem. Res..

[9] Nomikos P., MacGregor J.F. (1994). Monitoring Batch Processes Using Multiway Principal Component Analysis. AIChE J..

[10] Nomikos P., MacGregor J.F. (1995). Multivariate SPC Charts for Monitoring Batch Processes. Technometrics.

[11] Nomikos P., MacGregor J.F. (1995). Multi-Way Partial Least Squares in Monitoring Batch Processes. Chemom. Intell. Lab. Syst..

[12] James G., Witten D., Hastie T., Tibshirani R. (2013). An Introduction to Statistical Learning.

[13] Elkan C. (2001). The Foundations of Cost-Sensitive Learning. Proceedings of the International Joint Conference on Artificial Intelligence.

[14] Gan D., Shen J., An B., Xu M., Liu N. (2020). Integrating TANBN with Cost Sensitive Classification Algorithm for Imbalanced Data in Medical Diagnosis. Comput. Ind. Eng..

[15] Verbeke W., Olaya D., Berrevoets J., Verboven S., Maldonado S. (2020). The Foundations of Cost-Sensitive Causal Classification. arXiv.

[16] Ghatasheh N., Faris H., AlTaharwa I., Harb Y., Harb A. (2020). Business Analytics in Telemarketing: Cost-Sensitive Analysis of Bank Campaigns Using Artificial Neural Networks. Appl. Sci..

[17] Lobo A., Oliveira P., Sampaio P., Novais P., Omatu S., Mehmood R., Sitek P., Cicerone S., Rodríguez S. (2022). Cost- Sensitive Learning and Threshold-Moving Approach to Improve Industrial Lots Release Process on Imbalanced Datasets. Proceedings of the Distributed Computing and Artificial Intelligence, 19th International Conference, L’Aquila, Italy, 13–15 July 2022.

[18] Zhang H., Jiang L., Li C. (2021). CS-ResNet: Cost-sensitive Residual Convolutional Neural Network for PCB Cosmetic Defect Detection. Expert Syst. Appl..

[19] Alves F.F., Nogueira T.H., Ravetti M.G. (2022). Learning Algorithms to Deal with Failures in Production Planning. Comput. Ind. Eng..

[20] Frumosu F.D., Khan A.R., Schiøler H., Kulahci M., Zaki M., Westermann-Rasmussen P. (2020). Cost-Sensitive Learning Classification Strategy for Predicting Product Failures. Expert Syst. Appl..

[21] Verbeke W., Olaya D., Guerry M.A., Van Belle J. (2023). To Do or Not to Do? Cost-sensitive Causal Classification with Individual Treatment Effect Estimates. Eur. J. Oper. Res..

[22] Mählkvist S., Ejenstam J., Kyprianidis K. Consolidating Industrial Batch Process Data for Machine Learning. Proceedings of the Scandinavian Simulation Society.

[23] Bergstra J., Bengio Y. (2012). Random Search for Hyper-Parameter Optimization. J. Mach. Learn. Res..

[24] Pedregosa F., Varoquaux G., Gramfort A., Michel V., Thirion B., Grisel O., Blondel M., Prettenhofer P., Weiss R., Dubourg V. (2011). Scikit-Learn: Machine Learning in Python. J. Mach. Learn. Res..

[25] Breiman L. (2001). Random Forests. Mach. Learn..

